# Antimicrobial resistance in human populations: challenges and opportunities

**DOI:** 10.1017/gheg.2017.4

**Published:** 2017-05-10

**Authors:** S. Allcock, E. H. Young, M. Holmes, D. Gurdasani, G. Dougan, M. S. Sandhu, L. Solomon, M. E. Török

**Affiliations:** 1 Department of Medicine, University of Cambridge, Cambridge, UK; 2 Wellcome Trust Sanger Institute, Genome Campus, Hinxton, UK; 3 Department of Veterinary Medicine, University of Cambridge, Cambridge, UK; 4 Jeremy Coller Foundation, London, UK; 5 Cambridge University Hospitals NHS Foundation Trust, Cambridge, UK; 6 Clinical Microbiology and Public Health Laboratory, Public Health England, Cambridge, UK

**Keywords:** Animal, antimicrobial, human, multidrug-resistant, resistance

## Abstract

Antimicrobial resistance (AMR) is a global public health threat. Emergence of AMR occurs naturally, but can also be selected for by antimicrobial exposure in clinical and veterinary medicine. Despite growing worldwide attention to AMR, there are substantial limitations in our understanding of the burden, distribution and determinants of AMR at the population level. We highlight the importance of population-based approaches to assess the association between antimicrobial use and AMR in humans and animals. Such approaches are needed to improve our understanding of the development and spread of AMR in order to inform strategies for the prevention, detection and management of AMR, and to support the sustainable use of antimicrobials in healthcare.

## Introduction

Antimicrobial resistance (AMR) – the ability of microbes to grow in the presence of a drug that would normally kill them or limit their growth [1] – is a major global public health concern [2, 3]. AMR complicates the treatment of infection and is associated with increased morbidity and mortality. The emergence and spread of resistant and multidrug-resistant (MDR) bacteria has enormous implications for worldwide healthcare delivery and population health [4, 5]. The reporting of bacterial isolates that have transmissible resistance to carbapenems and colistin (considered to be the last-line antibiotics for MDR Gram-negative bacterial infections) emphasises this major public health threat [6]. Furthermore, the lack of development of new antimicrobials in over two decades adds to the urgency of preserving the antimicrobial efficacy of currently available drugs. Although the role of antimicrobial use in the development of AMR has been studied extensively, the emergence, distribution and transmission of AMR in populations is complex and poorly understood. AMR in populations is influenced by bacterial, host, and environmental factors, including exposure to antimicrobials in clinical medicine, environmental waste and contamination, food production and animal husbandry [7–9]. Here, we review the current understanding of mechanisms underlying AMR at the population and individual level, specifically highlighting gaps where further research is needed across all income settings. Understanding the determinants of AMR in the community will be fundamental in developing effective strategies to prevent and manage AMR.

## The burden of AMR

Antimicrobials have played a crucial role in reducing morbidity and mortality from infectious diseases. However, these healthcare gains are now under threat because of the emergence and spread of organisms that are resistant to antimicrobials [4, 5]. In recent decades, AMR has become a widespread problem in all countries, irrespective of their level of income. In 2014, a synthesis of evidence and economic review indicated that an estimated 700,000 deaths globally were attributable to infections caused by antibiotic-resistant organisms, and this is expected to reach 10 million/year by 2050 [4, 10, 11]. It has also been predicted that the mortality from infections in which AMR is a factor could result in a reduction of 2% to 3.5% in global gross domestic product in 2050, amounting to between 60 and 100 trillion US dollars, globally [4, 10, 11]. This economic cost may be more evident in low- and middle-income countries (LMICs); however, the impact of AMR on these LMICs remains largely unknown [12]. The costs associated with AMR are likely to increase as resistance to second- and third-generation antibiotics develops, leading to scenarios where critically ill patients need supportive care, and antibiotics no longer have therapeutic efficacy [13].

## Mechanisms of AMR

The development of AMR is primarily due to selective pressure on microorganisms – as a result of exposure to antimicrobials. There are several mechanisms by which organisms can adapt and become resistant to antimicrobials; these include the production of enzymes, alteration of target sites, alteration of metabolic pathways, alteration of outer membrane permeability and efflux pumps [14]. Resistant bacteria may possess one or more of these mechanisms, and thus exhibit resistance to more than one class of antimicrobial. Genetic variation is essential for microbial evolution, and may arise by a variety of mechanisms including point mutations, rearrangements of large segments of DNA from one location of a chromosome or plasmid to another, or acquisition of foreign DNA from other bacteria by horizontal transfer of mobile genetic elements. A single mutation that confers AMR in a bacterium in a population under selection pressure can enable survival of that organism where all other susceptible organisms are killed [15]. The resistant organisms can continue to replicate, becoming the dominant variant [15].

## AMR surveillance

The first step to tackling AMR is to determine the burden and understand its determinants before implementing public health policies to contain it. Whilst the USA and many European countries have surveillance systems in place, these are mostly lacking in low-resource settings. Surveillance of AMR in these regions is limited by the lack of laboratory infrastructure for monitoring infections and detecting resistance. Smaller studies examining the prevalence of AMR in these regions are likely to be hospital-based, and potentially overestimate the prevalence of AMR [2]. Development of national and regional collaborative surveillance networks with free data sharing may provide important frameworks for successful monitoring and reporting in these regions. Dissemination of trend information from such setups and engagement with policy-makers would be essential to devising regional strategies for containment of AMR. One example of such a network is the ReLAVRA (the Latin American Antimicrobial Resistance Surveillance Network) created in 1996 by the World Health Organization regional office to collect and aggregate data from National Reference Laboratories in the region [2]. This has greatly improved the ability to detect and monitor AMR, and has played a role in guiding policy towards antimicrobial use in this region. Transposing similar efforts to surveillance of AMR in other settings such as Africa and South-East Asia will be essential to developing a global response to AMR.

## Antimicrobial use and AMR in humans and other animals

The use of antimicrobials in clinical medicine has materially decreased the burden of infectious diseases and facilitated complex medical interventions such as organ transplantation and advanced surgery [5]. Antimicrobial use, misuse or overuse in clinical medicine is a major contributing factor in the development of AMR in human populations [3, 16–18]. Antimicrobials are also widely used in domestic animals and livestock [19]. Sub-therapeutic doses of antimicrobials are used for growth-promotion in some countries [20]. Although the use of antimicrobials as growth promoters has been discontinued in the European Union since 2006 [21], this practice continues in the Americas and Asia [22]. Current livestock husbandry systems lead to relatively high levels of endemic disease [19] but, in the case of pigs and poultry, it is often uneconomic to provide treatment at the individual animal level, leading to the blanket treatment of groups of animals, normally delivered via the feed or water [23]. Together with antimicrobial exposure in the environment, antimicrobial use in humans and other animals is thought to be an important selective pressure for AMR globally [19, 24]. Additionally, there are important gaps in our understanding of the role of food-borne bacteria, and their impact on health at the population level [2]. Implementing surveillance of AMR relating to food products would require integrated surveillance of AMR at hospital and community levels, including surveillance of AMR in food-producing animals [2]. Data surveillance networks at the national and global levels that could potentially identify and collect information promptly on index cases are necessary to better understand the impact of these factors.

## Transmission of AMR among human and animal populations

There is an intricate interplay between humans, animals and the environment in relation to the development and spread of AMR [7, 8, 25]. There have been many studies exploring the association between antimicrobial use in animals and resistance in humans, involving direct and indirect routes of transmission [19]. In 2015, the European Centre for Disease Prevention and Control, the European Food Safety Authority and the European Medicines Agency conducted an aggregated analysis of surveillance data from across the EU, assessing the relationship between human and animal use of antibiotics and AMR [26]. They reported that studies showed positive associations between consumption of antimicrobials and resistance in bacteria in both humans and animals. Some studies also found positive associations between antimicrobial consumption in animals and resistance in bacteria from humans. However, the aggregated European data also show varied or inconsistent associations among specific pathogens and AMR in humans and animals [26]. Whilst there are some studies that have shown that farm animals do not share common populations of some bacterial strains with humans [27], the emergence of livestock-associated methicillin-resistant *Staphylococcus aureus* (MRSA) such as clonal complex 398 (CC 398) [28] and *mecC* MRSA in a wide range of host species [29, 30] has shown that these antimicrobial-resistant lineages represent a common population shared by humans and farm animals. These assessments recapitulate the need to interpret such data with caution. By their nature, these aggregated data analyses of observational evidence are also limited by potential confounding factors [31, 32] – highlighting the need for more detailed and context-specific studies to examine the associations among antimicrobial exposure in humans and other animals, and the development of AMR.

The integration of genetic and epidemiological approaches, including targeted and whole-genome sequencing methods, has allowed the examination of the evolutionary origins of pathogens in both human and other animal populations. Analyses of sequence data suggest that a globally distributed MRSA lineage (ST5) may have originated after human-to-poultry transmission [33]. Conversely, studies have also shown that various MRSA lineages from animals also appear in humans [34, 35]. However, the direction of transmission (human-to-animal or animal-to-human) may be equivocal. Although there is evidence to indicate that third-generation cephalosporin-resistant *Escherichia coli* strains can spread from livestock to humans via food consumption [36, 37], a recent study suggested that such cephalosporin resistance genes are mainly disseminated in animals and humans via distinct plasmids [38]. Similarly, whole-genome sequencing analyses suggest that the multidrug-resistant *Salmonella* Typhimurium DT104 bacterium and its resistance genes are distinct between animal and human populations with limited transmission between the two [39]. Collectively, these studies highlight the complexities in understanding the burden and transmission of AMR among populations.

## The need for epidemiological surveillance using sequencing technologies

Despite growing worldwide attention, there are substantial limitations in our understanding of the burden, distribution and determinants of AMR at the population level. A recent systematic review of studies assessing the observational evidence for an association between antimicrobial use and development of MRSA resistance found that individual study reporting was poor [40]. This highlights the broader need for high-quality epidemiological evidence, including for populations at high risk as well as those that are globally representative. Population prevalence studies are important for understanding the burden of AMR in a given setting and for informing context-specific treatment practices [41]. Such studies, in parallel to those assessing the health impact of antimicrobial-resistant pathogens, would help to inform the development of preventative interventions. Importantly, augmenting existing surveillance programmes will be central to this objective.

With a lack of new therapeutic agents to target resistant bacterial infections, it is increasingly important to understand the factors that contribute to the emergence of AMR. While most preventative strategies emphasise the need to reduce antimicrobial misuse and overuse by health providers, the lack of advice regarding specific interventions that are likely to have an impact on reduction in AMR in the hospital and community setting limits the implementation of such guidance. Well-designed large-scale epidemiological studies are needed to understand the relationship between AMR and distinct patterns of antimicrobial prescribing and consumption, in order to inform specific guidelines for AMR stewardship in humans as well as agricultural treatment practices. Such research could also facilitate identification of high-risk populations (human and animal) for AMR transmission, and provide specific guidance for prevention and treatment in these subgroups. There is a need to assess this association in both hospital and community settings, including the use of routine clinical data, electronic health records (EHRs) and concomitant pathogen surveillance, as well as capitalising on experimental/interventional designs [42]. Potentially comparable data exist, but are limited because of a lack of standardised approaches for measuring resistance and harmonised surveillance and data collection guidelines. More data are needed to understand the complex relationship between antimicrobial use and consumption in many different settings, and the prevalence of AMR in humans and other animals. Integrated surveillance of AMR in food-producing animals, foods, and humans globally with standardised approaches and timely sharing of data is key to identifying potential routes and sources of transmission [2]. Sequencing-based approaches will play an important role in this endeavour [43]. These integrated approaches in human and animal populations will provide opportunities to help determine the evolutionary origins of pathogen lineages in both human and animal populations.

In addition to limitations of population-based epidemiological studies of AMR, there is also a gap in individual-level data to assess individual patient outcomes in response to antimicrobial use [7]. Existing systems, such as EHRs, could be utilised to help facilitate such studies, including the recruitment of individuals at the time of diagnosis of specific clinical indications, as well as documenting antibiotic treatment. EHRs could provide information on prescribing practices and the incidence of drug-resistant and MDR infections in hospital and community settings. Such an approach could provide strategies to assess the impact of empirical antimicrobial use in human populations and the prevalence of AMR. This may also enable a better understanding of the predictors of AMR, helping to identify and target emerging resistant infections. A critical element of such research will be to consider prospective designs (longitudinal studies), particularly in specific contexts – defined by geographical regions, communities and dominant pathways of potential zoonotic transmission. Such prospective designs would allow the real-time study of the development of resistant strains in the population, and allow a detailed examination of factors that encourage and impede the spread of AMR [42]. However, E-health systems have their limitations, including disparities in reporting and inconsistencies in completeness and reliability. Furthermore, whilst E-health systems have been established in a number of high-income settings, this is still a nascent field in LMICs. The utility of EHRs for epidemiological research into AMR first requires the implementation of E-health systems in LMICs, which will need to involve improved technical infrastructure and connectivity, technical capacity building and solutions to ensure sustainability and use of such systems. Where surveillance and clinical databases exist, these potentially provide a framework for epidemiological studies of AMR. However, these programmes will need to be expanded to include reporting of animal infections and AMR, as well as veterinary prescribing practices. Furthermore, sharing and accumulation of these data through a central repository could enable an assessment of this relationship on a national or regional scale.

## Prevention and treatment

As well as efforts to increase our understanding of the factors contributing to the emergence and spread of AMR, there are preventative strategies that could help to reduce its burden. Preventing the spread of infections in the first instance would reduce the need to use antimicrobials and, consequently, might moderate the development of resistant infections. There are a number of ways in which this might be achieved. Examples include immunisation, safe food preparation, infection control measures (e.g. hand hygiene, barrier precautions and isolation of infected patients) and improved waste management to prevent the spread of resistant organisms in the hospital, community, agricultural and environmental settings. However, basic health interventions, such as good hygiene practices and adherence to antimicrobial stewardship, can be particularly difficult to achieve in some LMICs [44]. Furthermore, human resource constraints and weak health infrastructures limit the surveillance and management of AMR [45, 46]. It will therefore be important to tailor AMR preventative measures to the local context in order to make them achievable and effective. The development of rapid diagnostic tests that could be used in both low- and high-resource settings to rapidly diagnose bacterial infection and identify markers of drug resistance would also be beneficial to this endeavour.

Patterns of antimicrobial consumption are influenced by multiple factors including self-diagnosis and treatment with over-the-counter medications and local prescribing practices. The lack of point-of-care (POC) tests that are able to reliably distinguish bacterial from viral infections means that patients are frequently prescribed empirical antimicrobials whilst waiting for the results of microbiological tests [47]. The dose or duration of treatment may also be sub-optimal, allowing the emergence of resistant strains. A lack of public understanding of AMR also influences patterns of consumption. Patients often discontinue antimicrobial treatment before the end of the treatment course, which supports the selection of antimicrobial resistant organisms [3]. Only half of countries have implemented a campaign for public education on the use of antimicrobials, and the majority of these countries are higher-income [48]. Furthermore, the availability and use of substandard or counterfeit antimicrobials adds to the challenges of antimicrobial stewardship, as well as risking lives [49, 50].

Increasing globalisation also contributes to the development and spread of AMR. Population movement facilitates the spread of resistant infections from areas of high to low prevalence. However, the risk of imported drug-resistant infections is difficult to quantify and better international surveillance is needed to detect and monitor these infections. Deployment of new technologies, such as next-generation sequencing, could help to better understand the origin of organisms, and the role of population movement in the spread of AMR. Furthermore, in LMICs that are undergoing rapid economic development, urbanisation has resulted in overcrowding and poor sanitation. Development may also result in a rise in migration from rural to urban areas. This not only increases exposure of susceptible populations to drug-resistant infections, but further contributes to the problem of overcrowding. Collectively, these factors associated with urbanisation increase the risk of the development and spread of AMR. Better control of the spread of AMR will rely on a multi-sectorial approach and the involvement of stakeholders on a global scale.

Antimicrobial stewardship programmes (ASPs) involve a number of interventions to promote prudent antimicrobial use and reduce AMR, through evidence-based treatment regimens. EHR systems have recently been shown to provide a successful framework for ASPs [51]. These programmes require indications and criteria for specific antibiotic use to be specified prior to use. They also prompt clinicians to rationalise or discontinue antibiotic therapy after 48–72 h, in response to microbiology results. Additionally, by the creation of order sets, software developers can direct antibiotic prescribing towards a preferred regimen, when one regimen is noted to be associated with higher AMR, based on EHR surveillance [51]. Such intervention depends on the availability of comprehensive ecological and epidemiological data on AMR locally, nationally and even globally. One barrier to widespread implementation of ASPs is the financial cost of training, particularly in LMICs [46]. However, ASPs have been shown to be cost-effective and to reduce costs in the long term [46]. ASPs should be implemented alongside other effective interventions such as hand hygiene, which has been shown to reduce antimicrobial resistant infections [52]. Furthermore, across all income settings it will be important to promote integration with other programmes for pharmacy management, microbiology, POC diagnostics and laboratory quality control [46].

The advancement of precision medicine will provide additional opportunities to reduce the emergence and spread of AMR. On the one hand, it will enable the delivery of more targeted therapy against the infective organism. On the other hand, it may help to predict to susceptibility to infection, severity of disease or response to treatment in the infected individual. The application of a precision medicine approach may also help to reduce unnecessary antimicrobial prescribing and thus slow the emergence and spread of resistance. However, this approach often requires the availability of accurate, rapid and affordable diagnostic tests if we are to derive the potential health care benefits.

Finally, there is an urgent need to develop new therapeutic options for treating drug-resistant infections. Since 1987, no new class of antibiotics has been discovered and drug development has largely relied on structural changes to existing compounds [53, 54]. Challenges in target selection, especially targets that are essential, conserved and not susceptible to the rapid development of resistance, limit the success of antibiotic drug discovery and development. Further, the low risk of success and the high economic costs associated with drug development have become a major disincentive for pharmaceutical companies and resulted in many of them withdrawing from this field. Academic institutions are unable to take new antimicrobials beyond early stage development without industrial partners. Any new antimicrobials that are developed for the treatment of resistant and MDR infections will need to be highly restricted in their use, further reducing incentive for commercial drug development. A paradigm shift towards a new model for drug development that brings together academia, pharmaceutical companies and governments to share the risks and costs of drug development for public benefit is required. This solution has been proposed by the O'Neill Review on AMR [55].

## Conclusion

AMR is a global public health threat. Despite growing worldwide attention to AMR, there are substantial limitations in our understanding of the burden, distribution and determinants of AMR at the population level. The integration of a broad range of context-specific epidemiological study designs is needed to improve our understanding of the burden of AMR in populations, as well as the population-level factors influencing the development and spread of AMR [32]. Together with stewardship initiatives, such studies will better inform strategies for the detection, prevention and management of AMR and to support the continued use of antimicrobials in healthcare.
