# Antimicrobial resistance in human populations: challenges and opportunities – ERRATUM

**DOI:** 10.1017/gheg.2017.12

**Published:** 2017-10-27

**Authors:** S. Allcock, E. H. Young, M. Holmes, D. Gurdasani, G. Dougan, M. S. Sandhu, L. Solomon, M. E. Török

In the above publication [1], the contact details for corresponding author Dr Lulit Solomon were omitted. This error has now been rectified in the original article. The publisher apologises for this error.
